# An Automated, Online Feasibility Randomized Controlled Trial of a Just-In-Time Adaptive Intervention for Smoking Cessation (Quit Sense)

**DOI:** 10.1093/ntr/ntad032

**Published:** 2023-04-14

**Authors:** Felix Naughton, Aimie Hope, Chloë Siegele-Brown, Kelly Grant, Garry Barton, Caitlin Notley, Cecilia Mascolo, Tim Coleman, Lee Shepstone, Stephen Sutton, A Toby Prevost, David Crane, Felix Greaves, Juliet High

**Affiliations:** Behavioural and Implementation Science Group, School of Health Sciences, University of East Anglia, Norwich, UK; Addiction Research Group, Norwich Medical School, University of East Anglia, Norwich, UK; Behavioural and Implementation Science Group, School of Health Sciences, University of East Anglia, Norwich, UK; Addiction Research Group, Norwich Medical School, University of East Anglia, Norwich, UK; Department of Computer Science and Technology, University of Cambridge, Cambridge, UK; Norwich Clinical Trials Unit, University of East Anglia, Norwich, UK; Norwich Clinical Trials Unit, University of East Anglia, Norwich, UK; Addiction Research Group, Norwich Medical School, University of East Anglia, Norwich, UK; Department of Computer Science and Technology, University of Cambridge, Cambridge, UK; Centre for Academic Primary Care, University of Nottingham, Nottingham, UK; Norwich Clinical Trials Unit, University of East Anglia, Norwich, UK; Behavioural Science Group, University of Cambridge, Cambridge, UK; Nightingale-Saunders Clinical Trials and Epidemiology Unit, Kings College London, London, UK; Department of Behavioural Science and Health, University College London, London, UK; Department of Primary Care and Public Health, School of Public Health, Imperial College London, London, UK; Norwich Clinical Trials Unit, University of East Anglia, Norwich, UK

## Abstract

**Introduction:**

Learned smoking cues from a smoker’s environment are a major cause of lapse and relapse. Quit Sense, a theory-guided Just-In-Time Adaptive Intervention smartphone app, aims to help smokers learn about their situational smoking cues and provide in-the-moment support to help manage these when quitting.

**Methods:**

A two-arm feasibility randomized controlled trial (*N* = 209) to estimate parameters to inform a definitive evaluation. Smoker’s willing to make a quit attempt were recruited using online paid-for adverts and randomized to “usual care” (text message referral to NHS SmokeFree website) or “usual care” plus a text message invitation to install Quit Sense. Procedures, excluding manual follow-up for nonresponders, were automated. Follow-up at 6 weeks and 6 months included feasibility, intervention engagement, smoking-related, and economic outcomes. Abstinence was verified using cotinine assessment from posted saliva samples.

**Results:**

Self-reported smoking outcome completion rates at 6 months were 77% (95% CI 71%, 82%), viable saliva sample return rate was 39% (95% CI 24%, 54%), and health economic data 70% (95% CI 64%, 77%). Among Quit Sense participants, 75% (95% CI 67%, 83%) installed the app and set a quit date and, of those, 51% engaged for more than one week. The 6-month biochemically verified sustained abstinence rate (anticipated primary outcome for definitive trial), was 11.5% (12/104) among Quit Sense participants and 2.9% (3/105) for usual care (adjusted odds ratio = 4.57, 95% CIs 1.23, 16.94). No evidence of between-group differences in hypothesized mechanisms of action was found.

**Conclusions:**

Evaluation feasibility was demonstrated alongside evidence supporting the effectiveness potential of Quit Sense.

**Implications:**

Running a primarily automated trial to initially evaluate Quit Sense was feasible, resulting in modest recruitment costs and researcher time, and high trial engagement. When invited, as part of trial participation, to install a smoking cessation app, most participants are likely to do so, and, for those using Quit Sense, an estimated one-half will engage with it for more than 1 week. Evidence that Quit Sense may increase verified abstinence at 6-month follow-up, relative to usual care, was generated, although low saliva return rates to verify smoking status contributed to considerable imprecision in the effect size estimate.

## Introduction

The total number of smokers worldwide is increasing,^1^ making the delivery of effective and scalable cessation support crucial to reduce the estimated 8 million annual premature deaths because of tobacco.^2^ One current gap in cessation treatment is effectively supporting smokers to manage situational triggers to smoke.

Any smoking early in a quit attempt (a “lapse”) is highly predictive of longer-term return to smoking (“relapse”),^3,4^ including when experimentally manipulated.^5^ While data are limited, evidence suggests that approximately half of the lapses are induced by cues to smoke from an individual’s environment or setting,^6^ via “cue-induced cravings.” Cues that generate cravings can be “proximal,” for example, seeing unlit cigarettes or other people smoke, or “distal,” that is, the actual environments an individual has smoked in,^7,8^ with exposure to both types simultaneously intensifying cravings further.^8^ Helping smokers manage both proximal and distal cues could prevent early lapses and increase successful smoking cessation.

There is a lack of effective support to help smokers manage cue-induced cravings. Steady-state medications such as bupropion, Varenicline, and nicotine patches, do not address cue-induced cravings.^9,10^ While fast-acting nicotine replacement therapy can help reduce these cravings,^9^ few nicotine replacement therapy users use these types of treatment.^11,12^ E-cigarettes can be used to address these cravings, in countries where they are not prohibited such as the United Kingdom, though many smokers do not want to use them exclusively and multiple barriers can reduce their successful uptake, including safety concerns, lack of satisfaction, and device complexity.^13^ Cognitive or behavioral lapse prevention strategies, such as self-talk or avoiding smokers, can help smokers avoid or manage cue-induced cravings,^9,14,15^ though most strategies smokers spontaneously use are typically those with the least strong evidence base.^16^ An additional challenge is that the window of opportunity for intervening during a cue-induced craving is short. One of the only studies of its kind found half of lapses brought about by acute craving episodes occurred on average within 11 minutes after craving onset.^6^ Support to address cue-induced cravings, therefore, needs to be rapid, easily accessible in different locations, and deliver evidence-based strategies.

Meta-analyses pooling smoking cessation smartphone app evaluations have yet to demonstrate a benefit,^17^ although some cessation app evaluations have shown evidence of their effectiveness.^18^ Mobile device interventions that deliver support at random or prespecified times are referred to as just-in-time interventions and those which adapt this support to a real-time need or opportunity, are Just-In-Time Adaptive Interventions.^19,20^ Two main types of JITAIs, relevant for addictive behaviors, have emerged. Those using active measurement to identify real-time need, typically using self-completion ecological momentary assessment, and those using passive measurement, via on-board or wearable sensors or other data streams, or a combination.^21^ There are likely pros and cons to each of these approaches. Given the rapid time to lapse after the onset of craving, and evidence that some user-initiated, on-demand craving tools are seldom used beyond a first try,^22,23^ active measurement approaches may miss key instances of cue exposure in real time. In addition, there may be ecological momentary assessment disengagement as a result of measurement burden. On the other hand, an entirely passive measurement approach could struggle to infer lapse risk because of measurement error and exclude the smoker from learning to identify high-risk cues and situations and so miss out on the potential benefits of this and self-monitoring.^19^

The Quit Sense app was developed as a passive measurement JITAI for smoking cessation to address the gap in support for managing situational cues to smoke. It is trained by the smoker before their quit attempt starts so the app, and the smoker, can learn their smoking habits, including the locations where they smoke and the smoking cues which precede their smoking whilst they are within these locations. Then, once their quit attempt starts, Quit Sense delivers behavioral support triggered by and tailored to users’ smoking locations and their associated smoking cues. Hence, Quit Sense succeeds in providing “in the moment” support to smokers, including the provision of lapse prevention strategies, and is both engaged with and found to be acceptable.^7^ Quit Sense has been developed as a “native app,” meaning it does not need an internet connection to operate or need to share location or other data if deployed outside of research, maximizing privacy. While one small randomised controlled trial has been completed investigating an active measurement-based cessation JITAI,^24^ none have investigated a passive measurement or “context aware” JITAI such as Quit Sense. The current study was undertaken to establish the feasibility of a future definitive evaluation of Quit Sense and to provide an estimate of its impact on smoking cessation.

## Methods

### Design

A two-arm parallel randomized controlled feasibility trial, allocating smokers recruited online (1:1 ratio) to a “usual care” arm (referral to NHS SmokeFree website) or an intervention arm who received “usual care” plus an invitation to install the Quit Sense app. A nested qualitative process evaluation and a Study-Within-A-Trial (SWAT), evaluating retention incentives, were also undertaken though reported elsewhere. For full details of study methods, see the trial protocol.^25^

Ethical approval was received by the Wales REC7 NHS Research Ethics Committee (19/WA/0361) and a trial steering committee with a majority of independent members oversaw trial conduct. The trial was preregistered (ISRCTN12326962).

### Participants

Inclusion criteria were: A current smoker; aged 16 years and above; smoked at least 7 cigarettes per week; willing to make a quit attempt within 14 days of enrollment; own an Android smartphone (version 5.0 or above); a resident in England; able to provide informed consent; not having previously participated in the trial.

#### Sample Size.

Sample size was based on achieving adequate precision for key “full trial” parameters. In line with guidance on feasibility trials with binary outcome measures,^26^ a sample size of 100 per group was chosen, which for a primary self-reported smoking outcome completion of 80%^27,28^ provided precision (defined as the 95% confidence interval half-width) of +/− 6%.

### Procedure

Recruitment took place through paid-for online adverts with Facebook (including Instagram) and Google Search, limited to England-based IP addresses and targeted at Android devices. Online adverts were managed and optimized by a partner company called Nativve. Advert campaigns were run in two phases; from November 27, to December 12, 2020 and from January 5, to January 25, 2021. It was preplanned that if, after the first phase of recruitment or 35% of the target sample was reached, less than 45% of the sample were categorized as low socioeconomic status (SES) then over-sampling using advert targeting would be undertaken to increase low SES representation. Low SES was defined using the National Statistics Socio-Economic Classification,^29^ as individuals with a semi-routine or routine and manual occupation, class five in the National Statistics Socio-Economic Classification, or who have never worked or are long-term unemployed. Facebook targeting was also used to increase the ethnic diversity of the sample.

Clicking a study advert took individuals to the study website with study information including a downloadable participant information sheet. Those interested completed a screening survey and, if eligible, were asked to provide consent to participate using an e-signature. REDCaptcha, a captcha module available in REDCap,^30^ was used to prevent “bot” submissions. Participants then completed an online baseline questionnaire and afterward were randomly allocated to either the usual care or Quit Sense app arm. To promote study engagement, as recommended by our public engagement panel, all participants were sent a study text message 5 days post-enrollment thanking them for their involvement and after 12 weeks a postcard (in an envelope) with study summary information.

#### Randomization.

Randomization was stratified by smoking rate (<16 vs. ≥ 16 cigarettes/day; based on mean smoking rates from similar trials)^27,31^ and socioeconomic status (low vs. other categories). Allocation sequences were generated by computer-based random permuted blocks (varying block sizes) (using REDCap^30^). Randomization was integrated into the study website, therefore the sequence was concealed from participants until assignment and concealed from members of the trial team, other than the statistician, developers of the study database, and the lead researcher who was unblinded for potentially providing app installation support and selecting participants to interview as part of the qualitative process evaluation.

### Interventions

#### Usual Care.

After randomization, both arms received an automated text message with a link to the NHS SmokeFree website (www.smokefree.nhs.uk), and, if requested, also by email. At the time the trial was run, this website provided access and signposting to digital, telephone, and in-person cessation support in England.

#### Quit Sense App.

Quit Sense arm participants also received an automated text message providing a link to the Quit Sense app on the Google Play store, along with a unique activation code. If the app had not been installed after 3 days, participants were sent a reminder text message to encourage installation. Five days later, if still not installed, a further text message was sent inviting participants to reply by selecting one of the five pre-specified reasons for not having installed the app.

Quit Sense, a context-aware Just-In-Time Adaptive Intervention (JITAI), is informed by learning theory and two theory-guided SMS text message systems,^23,32^ which are informed by Social Cognitive Theory.^33^ Twenty-one Behavior Change Techniques^34,35^ are used to target eight theory-based determinants (see Supplementary Figure 1 for logic model).^25^

A central feature is “Geofence-Triggered Support” (GTS), which is orientated around three stages within the app:

Stage 1 (“train the app”): Using a real-time smoking reporting tool, the user trains the app to learn about their smoking behavior until their quit date arrives. Users are asked to report smoking using this tool each time they smoke. For each report, the user indicates the situational context when they “light up” (stress, mood,^36^ urge strength,^37^ situations [home, work, working from home, socializing, and other], and presence of other smokers), while the app records geolocation using location sensors. Feasibility work found the median smoking report completion time was 13 seconds.^7^ The app creates geofences (circular virtual perimeters) around each location where smoking is reported more than once. After each smoking report, tailored feedback and support are provided on screen.

Stage 2 (“commit to quit” – a 28-day abstinence challenge): After their quit date has passed, the app monitors the user’s location. If they enter and remain in (≥5 minutes) a smoking geofence, the app will determine whether to trigger a GTS message, based on smoking reporting history (using frequency thresholds) and time of day, for that location. GTS messages are tailored to the situation and many are also tailored using the context information from the smoking reports. GTS messages provide lapse prevention support to help manage or avoid potential cue-induced cravings. Further decisions about whether to trigger messages are made after each 3-hour interval of remaining in that location (default between 8:00 AM and 9:30 PM or defined by the user). New geofences are created if lapses are reported, as with stage 1.

Stage 3 (“maintain the change”): The app continues to deliver GTS for 2 further months but reduces the frequency by one-half every month. After 3 months post-quit date, the GTS support stops, unless the user opts to restart their quit attempt, which they can do at any time.

Quit Sense had additional features, relevant to all stages unless specified (for details see protocol^25^):

- An End of Day survey with feedback messages after completion.
- A “my profile” section providing days quit and money saved, a self-monitoring calendar showing emoji feedback for smoking, cravings, and self-efficacy for each end-of-day survey and smoking pattern graphical and written feedback for reported smoking triggers.
- A library of cessation advice messages across key topics.
- Scheduled morning daily support messages oriented around the quit date.
- Quit date reset option, either manually triggered or offered if relapse (more than one smoking episode reported each day over 2 consecutive days) is determined.

### Measures

At 6 weeks, participants were sent a text message with a questionnaire link, 1 day after receiving a prenotification text message alerting them to the questionnaire link message and a texted £5 Amazon voucher for completing it. A reminder text message was sent if the survey was not completed 4 days later, after which participants were informed a researcher would call to complete it over the phone. The researcher made up to five contact attempt episodes.

Six and a half months after enrollment (“6-month follow-up”), the same procedure as for the 6-week questionnaire was undertaken for the 6-month questionnaire, with participants randomized to receive either a £10 or £20 Amazon voucher incentive for completion, as part of the SWAT (with trial arm included as a stratifier to ensure the balance between incentive groups), reported elsewhere. If manual follow-up by telephone was unsuccessful, participants were sent a text inviting a response to the primary smoking outcome question. Participants reporting 7-day abstinence at 6 months were sent a postal saliva test kit and texted a £5 voucher incentive by the lead researcher if returned to the testing laboratory.

#### Feasibility Outcomes.

Feasibility outcomes used to estimate key parameters to inform a future trial included:

- Completeness of the anticipated primary outcome for a future definitive trial (see smoking outcomes).
- Abstinence rate of usual care arm (anticipated primary outcome).
- Advertising cost per recruit.
- Rates of app installation, use, and acceptability (recommend Quit Sense to a friend and ease of use [5-point scale]).
- Completion of smoking cessation-related resource use, including usual care use, and quality of life (EQ-5D-5L)^38^ data.
- Hypothesized mechanisms of action of Quit Sense (see smoking outcomes).

#### Smoking and Related Outcomes.

As a feasibility trial, there was no primary outcome, but the anticipated primary abstinence outcome for any future definitive trial was based on the Russell standard^39^: Self-reported abstinence in the previous 6 months allowing for no more than five cigarettes and not smoking in the previous week, biochemically validated by a saliva cotinine concentration of less than 10 ng/ml^39,40^ and for those using any nontobacco nicotine substitution, an anabasine concentration of less than 0.2 ng/ml.^40^ We also measured 7-day point prevalence abstinence at 6 weeks (self-report) and 6 months (self-report and biochemically verified), in line with recommendations.^41^

Hypothesized mechanisms of action collected at 6 weeks included lapse incidence in the first 2 weeks of a quit attempt or since enrollment if no attempt was made, mean frequency of use category (0, 1–5, 6–10, and >10 times) across 20 lapse prevention strategies for avoiding or coping with the desire to smoke,^16^ smoking cessation self-efficacy,^32^ Strength and Frequency of Urges To Smoke^36^ and automaticity and associative processes subscales from the Wisconsin Inventory of Smoking Dependence Motives (WISDM-37).^42^

### Analysis

Feasibility outcomes are described as proportions or summary statistics with 95% CIs. To estimate the intervention effect on abstinence, lapse incidence, and use of lapse prevention strategies, we used multiple logistic regression, providing ORs with 95% CIs, while adjusting for stratification variables and any prognostic covariates (baseline variables that are known to affect the outcome and may be unbalanced between trial arms), as defined by the prespecified statistical analysis and health economics plan (SHEAP; https://osf.io/mt6s5/). For the estimated intervention effect on abstinence, we assumed withdrawn or missing = smoking.^39^ Because of the relatively small number of abstinent participants, analyses using exact inference are also presented. The abstinence rate was also translated into interpretable probabilities using the Bayesian approach relevant to preliminary trials.^43^ This produces estimates of the probability that the underlying odds ratio (OR) is 1.7 or higher, or at other plausible values used to power a subsequent trial such as 1.5 or higher, or 2.0 or higher.

Between-group analyses of mechanisms of action variables were undertaken with the 2-sample *t*-test for continuous variables reasonably satisfying the normality assumption, and the Chi-squared or Mann–Whitney test otherwise, for categorical variables.

Sensitivity analyses of abstinence at 6 months were conducted excluding withdrawals, as well as a complete case analysis. Although planned, a missing data analysis using the full information maximum likelihood method was not technically possible in commonly available statistical software.

## Results

**Figure 1.**
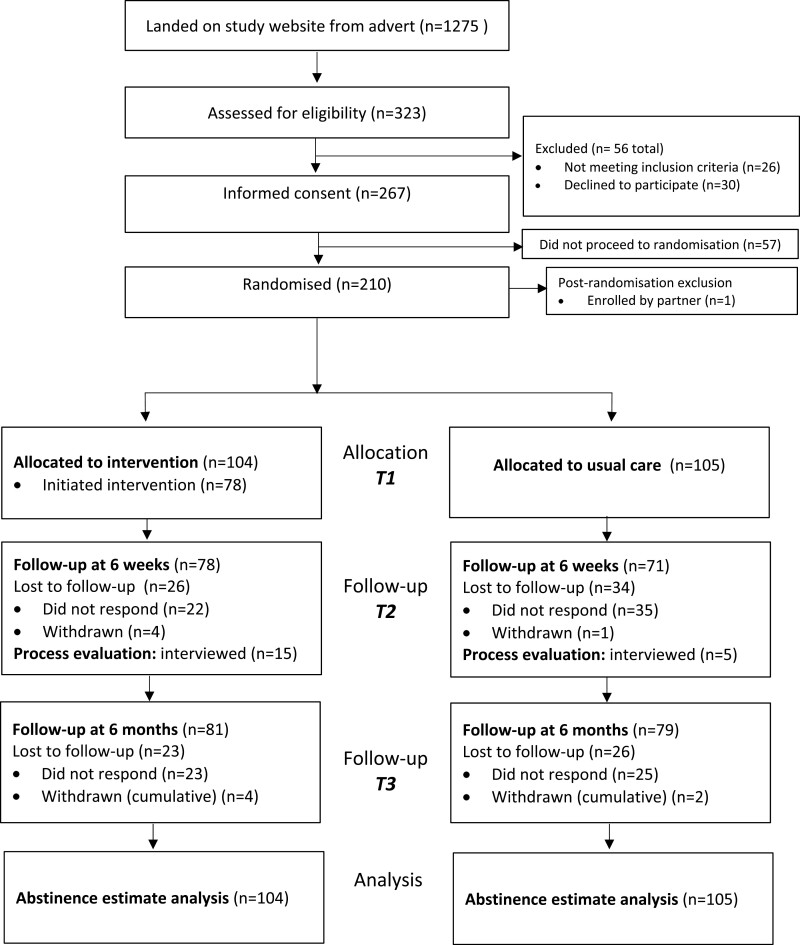
Trial flow.

There were 1275 study website landings from the online adverts, of which 323 (25%) people completed the eligibility assessment. Of those assessed for eligibility, 93% (299) were eligible and of those eligible, 70% (209) consented and were randomized (117 in phase one and 92 in phase two). One additional individual was randomized but had been enrolled by their partner and so was removed. See Figure 1 for trial flow. This left 104 allocated to the Quit Sense arm and 105 to usual care.

Table 1 provides baseline sample characteristics. The sample had a mean age of 41 years (range 18–61), 56% female, 29% classified as low socioeconomic status, 9% of nonwhite ethnicity, and mean baseline smoking rate of 15 cigarettes per day.

**Table 1.** Participant Characteristics at Baseline

	Quit Sense (*n* = 104)	Usual care (*n* = 105)	Overall (*n* = 209)
Age at consent: mean (SD), range	39.6 (10.0)	42.6 (10.0)	41.1 (10.0)
Gender: *n* (%)			
Male	45 (43.3%)	48 (45.7%)	93 (44.5%)
Female	59 (56.7%)	57 (54.3%)	116 (55.5%)
Number of cigarettes smoked per day: *n* (%)			
Less than 16	64 (61.5%)	63 (60.0%)	127 (60.8%)
16 or more	40 (38.5%)	42 (40.0%)	82 (39.2%)
Socioeconomic status: *n* (%)			
Low	30 (28.9%)	31 (29.5%)	61 (29.2%)
High	74 (71.2%)	74 (70.5%)	148 (70.8%)
Socioeconomic grade: *n* (%)			
1 (highest)	48 (57.1%)	46 (65.7%)	94 (61.0%)
2	8 (9.5%)	5 (7.1%)	13 (8.4%)
3	4 (4.8%)	7 (10.0%)	11 (7.1%)
4	13 (15.5%)	8 (11.4%)	21 (13.6%)
5 (lowest)	11 (13.1%)	4 (5.7%)	15 (9.7%)
Missing	20	35	55
*Ethnicity*: *n* (%)			
White	95 (91.4%)	96 (91.4%)	191 (91.4%)
Indian	0 (0.0%)	1 (1.0%)	1 (0.5%)
Pakistani	2 (1.9%)	0 (0.0%)	2 (1.0%)
Bangladeshi	1 (1.0%)	2 (1.9%)	3 (1.4%)
Black African	1 (1.0%)	1 (1.0%)	2 (1.0%)
Black (Other)	1 (1.0%)	0 (0.0%)	1 (0.5%)
Asian	2 (1.9%)	1 (1.0%)	3 (1.4%)
Mixed Race	1 (1.0%)	2 (1.9%)	3 (1.4%)
Not given	1 (1.0%)	2 (1.9%)	3 (1.4%)
Number of cigarettes usually smoke a day: Mean (SD)	15.4 (7.6)	15.5 (6.5)	15.4 (7.1)
Employment status: *n* (%)			
In work during last 12 months	83 (79.8%)	70 (66.7%)	153 (73.2%)
Out of work for more than 12 onths	19 (18.3%)	27 (25.7%)	46 (22.0%)
Retired	1 (1.0%)	0 (0.0%)	1 (0.5%)
Full-time student	1 (1.0%)	8 (7.6%)	9 (4.3%)
Occupation: *n* (%)			
Modern professional	22 (26.2%)	17 (24.3%)	39 (25.3%)
Clerical	9 (10.7%)	9 (12.9%)	18 (11.7%)
Senior manager/administration	11 (13.1%)	11 (15.7%)	22 (14.3%)
Technical	7 (8.3%)	8 (11.4%)	15 (9.7%)
Semi-routine manual/service	13 (15.5%)	6 (8.6%)	19 (12.3%)
Routine manual/service	7 (8.3%)	2 (2.9%)	9 (5.8%)
Middle/junior manager	6 (7.1%)	10 (14.3%)	16 (10.4%)
Traditional professional	9 (10.7%)	7 (10.0%)	16 (10.4%)
Missing	20	35	55
Highest Qualification: *n* (%)			
No formal	6 (5.8%)	7 (6.7%)	13 (6.2%)
GCSE or similar	22 (21.2%)	20 (19.1%)	42 (20.1%)
A/AS Level or similar	24 (23.1%)	28 (26.7%)	52 (24.9%)
Degree or similar	46 (44.2%)	44 (41.9%)	90 (43.1%)
Other	6 (5.8%)	6 (5.7%)	12 (5.7%)
Heaviness of smoking index: *n* (%)			
Low	36 (34.6%)	29 (27.6%)	65 (31.1%)
Moderate	57 (54.8%)	67 (63.8%)	124 (59.3%)
High	11 (10.6%)	9 (8.6%)	20 (9.6%)
Frequency of urges to smoke (FUTS): *n* (%)			
A little of the time	12 (11.5%)	5 (4.8%)	17 (8.1%)
Some of the time	29 (27.9%)	39 (37.1%)	68 (32.5%)
A lot of the time	39 (37.5%)	47 (44.8%)	86 (41.2%)
Almost all the time	17 (16.4%)	6 (5.7%)	23 (11.0%)
All the time	7 (6.7%)	8 (7.6%)	15 (7.2%)
Strength of urges to smoke (SUTS): *n* (%)			
No urges	1 (1.0%)	0 (0.0%)	1 (0.5%)
Slight urges	5 (4.8%)	6 (5.7%)	11 (5.3%)
Moderate urges	36 (34.6%)	49 (46.7%)	85 (40.7%)
Strong urges	38 (36.5%)	31 (29.5%)	69 (33.0%)
Very strong urges	20 (19.2%)	14 (13.3%)	34 (16.3%)
Extremely strong urges	4 (3.9%)	5 (4.8%)	9 (4.3%)
EQ-5D-5L utility score: mean (SD)	0.79 (0.22)	0.77 (0.21)	0.78 (0.22)
Missing	1	0	1
WISDM Automaticity subscale score: mean (SD)	4.71 (1.87)	4.79 (1.62)	4.75 (1.75)
WISDM Associative processes subscale score: mean (SD)	4.70 (1.34)	4.50 (1.34)	4.60 (1.34)
Self-efficacy score: mean (SD)	1.69 (0.92)	1.65 (0.81)	1.67 (0.87)

WISDM = Wisconsin Inventory of Smoking Dependence Motives.

At 6 weeks, 149 (71%; 95% CI 65%, 77%) were followed up and at 6 months this was 160 (77%; 95% CI 71%, 82%). There were six withdrawals, four from the Quit Sense arm and two from the usual care arm. Completion of self-reported abstinence for the primary outcome at 6 months was 77% (160/209; 95% CI 71%, 82%)). By arm, response rates were 78% and 75% for the Quit Sense and usual care arms, respectively. The return of a viable saliva sample for biochemical validation of those self-reporting abstinence for the primary outcome was 39% (16/41; 95% CI 24%, 54%), and by arm 52% (13/25) and 19% (3/16) for Quit Sense and usual care arms respectively. At the 6-month follow-up, the response rate for both resource use and quality of life data were 147/209 (70%; 95% CI 64%, 77%).

Advertising running costs were £2796 and advert costs were £804.44 for Facebook and £412.49 for Google search (grand total £4012.93). Total cost per recruit was £19.20 (US$23.80) (£13.38 running costs, £5.82 advert costs). 195 participants were recruited via Facebook and 14 from Google, with lower advert costs per recruit for Facebook (£4.13) than Google (£29.46). See Supplementary Figure 2 for further details. The higher proportion of nonwhite participants in phase 2 (13.8%) relative to phase 1 (5.2%) suggested targeting for nonwhite ethnicity may have been effective though this was not the case for targeting low SES participants (phase 1 30.4% vs. phase 2 29.9%).

The installation rate of the Quit Sense app, defined as receipt of the unique code provided to intervention participants by the app’s server, was 75% (95% CI 67%, 83%; 78/104) (Table 2). All but one participant (99%; 77/78) who installed the app did so before the installation text message reminder was sent. Several participants who installed the app (*n* = 9) did not have engagement data uploaded to the server because of a technical issue. In some of these cases, missing engagement data were determined from follow-up questionnaires or during process evaluation interviews. Among those who installed the app, 100% (95% CI 95%, 100%; 70/70) set a quit date in the app and 51% (95% CI 39%, 63%; 38/74) engaged with the app for more than 7 days, and 23% for more than 30 days (95% CI 13%, 33%), with a median duration of use of 10 days (IQR 30). Among installers who engaged with the app until their quit date (36/74; 49%), the total median duration of app engagement was 27 days (IQR 91). Among intervention participants who installed the app and were followed up at 6 weeks, 67% (29/46) said they would recommend Quit Sense to a friend trying to quit with 30% (13/46) saying “maybe” and 2% (1/46) that they would not recommend it. Most participants either strongly agreed (55%; 24/44) or agreed (32%; 14/44) that Quit Sense was easy to use.

**Table 2.** Use of Quit Sense app (Quit Sense arm only)

	Quit Sense arm
*Data uploaded to server from Quit Sense app*	
Participants that installed the app *n* (%)	78 (75.0%)
Duration of app use (days): median (q_25_,q_75_); minimum, maximum (*N* = 71^a^)	10 (1, 31); 0, 261
Number of active engagements: median (q_25_,q_75_), maximum, minimum (*N* = 69^a^)	11 (2, 39); 0, 271
Participants that engaged with the app for more than 7 days (*N* = 74^a^)	38 (51.4%)
Participants engaging with the app for more than 30 days (*N* = 74^a^)	17 (23.0%)
Participants that set a quit date in the app (*N* = 70^a^)	70 (100%)
Number of app quit dates per participant: median (q_25_,q_75_), maximum, minimum (*N* = 74^a^)	1 (1, 1); 0, 8
Participants that engaged with the app up until their quit date (of those that set a quit date; *N* = 74^a^)	36 (48.6%)
*Self-reported data at follow-up*	
Participants reported they would recommend Quit Sense to a friend trying to quit: *n* (%) (*N* = 43^a^^,^^b^)	
Unsure	0 (0.0%
No	1 (2.3%)
Maybe	13 (30.2%)
Yes	29 (67.4%)
Missing	31
Participants reported they found the app easy to use (4 or 5 on 5-point scale) (*N* = 44^a^^,^^b^): *n* (%)	38 (86.4%)

^a^A subgroup of participants (*n* = 9) experienced technical issues meaning recorded data was not uploaded from the app to the server and so is unknown. The number of these participants who were excluded from estimates varies as in some cases missing data was obtained via follow-up questionnaire or qualitative interview.

^b^Excluding those that did not install the app, were lost to follow-up or withdrew.

Analysis of the primary smoking outcome, which we anticipated would be used in a future study, found a higher abstinence rate in the Quit Sense arm (11.5%; 12/104) compared to the usual care arm (2.9%; 3/105) (unadjusted OR: 4.44, 95% CI 1.21, 16.21, *p* = .024) (Table 3). When adjusting for stratification variables and prognostic factors (heaviness of smoking index), there were no meaningful changes to the effect estimate (adjusted OR: 4.46, 95% CI 1.19, 16.69, *p* = .023). Because of relatively few abstinent participants, the model fit for the adjusted analysis was potentially problematic. The exact inference analysis produced more conservative (wider confidence intervals), but overall consistent results and conclusions (adjusted OR: 4.36, 95% CI 1.10, 25.22, *p* = .033). Sensitivity analyses were undertaken where (1) withdrawals were excluded and (2) complete cases were only included, using exact inference. Other than changing the abstinence proportions, the results remained consistent (see Supplementary Tables 1 and 2).

**Table 3.** Between-Arm Differences in Abstinence for Smoking Outcomes

Outcome	App group *n* (%) *N* = 104	Usual care *n* (%) *N* = 105	Difference % (95% CI)	Unadjusted OR^a^^,^^b^ (95% CI)	*p*-value	Adjusted OR model 1^c^ (95% CI)	Adjusted OR model 2^c^^,^^e^ (95% CI)
6-month prolonged abstinence validated by saliva test (primary)	12 (11.5%)	3 (2.9%)	8.7% (1.6%, 16.5%)	4.44 (1.21, 16.21)	0.024	4.46^d^ (1.19, 16.69)	4.36 (1.10, 25.22)
7-day point prevalence abstinence at 6 months validated by saliva test	16 (15.4%)	5 (4.8%)	10.6% (2.4%, 19.2%)	3.64 (1.28, 10.33)	0.015	3.67^d^ (1.27, 10.60)	3.59 (1.18, 13.18)
7-day point prevalence abstinence at 6 months, self-report	28 (26.9%)	20 (19.1%)	7.9% (−3.6%, 19.1%)	1.57 (0.82, 3.01)	0.178	1.54 (0.80, 2.97)	1.53 (0.76, 3.11)
7-day point prevalence abstinence at 6 weeks, self-report	20 (19.2%)	21 (20.0%)	−0.8% (−11.5%, 10.0%)	0.95 (0.48, 1.89)	0.889	0.91 (0.45, 1.83)	0.91 (0.43, 1.93)

^a^OR = Odds ratio (the odds of abstinence for participants in the app group is [OR] times that of the odds of abstinence for participants in the standard group).

^b^95% Wald confidence Interval.

^c^Adjusted for differences in smoking rate at baseline, socioeconomic status at baseline (stratification variables), heaviness of smoking index category at baseline (prognostic variable) and treatment group.

^d^Quasi-complete separation (model fit questionable) because of low cell counts in heaviness of index categories.

^e^95% Exact confidence Interval for OR.

By using the Bayesian approach relevant in preliminary trials, it is estimated there is 90% probability that the underlying OR favoring the intervention is 1.7 or higher, 93% that it is 1.5 or higher, and 85% that it is 2.0 or higher, indicating good support for a subsequent trial in which this range of effect sizes is considered.

Because of imbalanced saliva sample return rates between arms, we undertook a post hoc sensitivity analysis for the primary smoking outcome but using self-reported prolonged abstinence only. The findings favored the Quit Sense arm, though the between-arm difference was not statistically significant (Quit Sense 24.0%; usual care 15.2%, OR: 1.76, 95% CI 0.88, 3.53, *p* = .11). Other smoking outcomes at 6 months also favored Quit Sense over usual care, although this was only statistically significant for validated 7-day point prevalence (Table 3). Self-reported 7-day point prevalence abstinence at 6 weeks did not favor either arm.

At 6 weeks of follow-up, 70.4% in the Quit Sense arm and 80.8% in the usual care arm reported smoking in the first 2 weeks of a quit attempt (or since enrollment if no quit date was set) which was not statistically significant (*χ*^2^, [1, 149] = [2.17], *p* = .14) (Supplementary Table 3). There was no evidence of a difference between arms on average lapse prevention strategy use (mean difference: −0.07, 95% CI −0.26, 0.12, T-statistic: −0.75, *p* = .46) or when broken down into avoidance or coping strategies. There was no evidence of between-arm differences in self-efficacy, strength or frequency of urges to quit, and WISDM automaticity and associative processes subscale scores (Supplementary Table 3).

## Discussion

This was the first randomized controlled trial of a JITAI smartphone app for smoking cessation that uses passive measurement to trigger behavioral support. The automated online trial design employed was feasible and successful in reaching the target sample size within the relatively short recruitment timeframe. The cost per participant was compared favorably with other online digital cessation trials.^44,45^ Given the participants were exclusively identified online via adverts, the follow-up rate estimate was close to anticipated levels and is towards the higher end of rates achieved in other web-based cessation trials with comparable samples in terms of mean age, gender, and education.^28^

The trial also demonstrated that three-quarters of smokers assigned to the Quit Sense app would install it on their phones and engage with it at least to the point of setting a quit date. Very few evaluations report the uptake rates for cessation apps and those that do have either offered incentives for installation^46^ or required app installation for study inclusion.^47^ Compared to the largest cessation app evaluation undertaken to date, for “iCanQuit,” which demonstrated effectiveness, Quit Sense participants had a higher median number of days of use (10 days; server recorded, vs. 6 days; self-report).^48^ Among approximately half of Quit Sense installers who remained engaged at least till their quit date, the median total engagement duration was substantively higher (27 days). Future work investigating approaches to optimally increase engagement with smoking cessation apps while enhancing commitment to making a quit attempt would potentially increase the effectiveness of these interventions.

Trial findings provided promising evidence that Quit Sense increased verified cessation at 6 months compared to usual care, though with considerable imprecision in line with a feasibility trial and due to low return rates of saliva samples. No between-arm differences were observed at 6 weeks of follow-up, suggesting any benefit from Quit Sense, relative to usual care, was more likely because of maintaining abstinence in the longer term rather than from increasing the proportion of participants initiating a quit attempt. However, there was no quantitative evidence that Quit Sense affected the hypothesized mechanisms of action at 6 weeks of follow-up. The qualitative process evaluation provided some insights into how Quit Sense was felt to bring about abstinence among those participants interviewed and is reported elsewhere.

### Strengths and Limitations

One innovation employed was those trial procedures, other than manual follow-up and saliva sample posting, were fully automated once setup, meaning study running costs were relatively low. Approximately half of the participants did not require any human involvement at any stage of their trial involvement and for those that did; it was mainly manual telephone follow-up. As the automated procedures were successful, few changes would be needed when running a definitive evaluation, further reducing resource need and the risk of recruitment and measurement issues.

Further strengths were applying robust randomization and intervention delivery fidelity, publishing protocols, and making the statistical and health economic analysis plan publicly accessible prior to analysis, embracing key principles of open science.^49^

A key limitation was the poor return rate of saliva samples and that this was imbalanced between arms. It is possible the overall response was affected by the coronavirus disease 2019 pandemic, for example, more limited access to postal services due to changes in movement and time spent outside of the home or hesitation to provide a sample. The low incentive of £5 for a returned sample is likely to have been a factor. Similar trials have provided higher incentives for saliva returns and achieved higher response rates, including digital cessation intervention trials of online smokers (£20 incentive, 75% response rate; personal communication)^27^ and pregnant smokers (£30 incentive, 70% return rate).^50^ The post hoc sensitivity analysis investigating the potential influence of a reporting bias for returning a saliva sample found a smaller and nonsignificant intervention effect, though still favoring the intervention arm and with a similar absolute between-arm difference as the primary analysis. Whether the lower return rate observed for the usual care arm was because of apathy or higher levels of abstinence misreporting, increasing the incentive in a future trial would be expected to substantially increase the response rate and reduce the risk of reporting bias. A further potential risk of bias was interviewing participants as part of the process evaluation around 6 weeks post-enrollment. This could have acted as an intervention and while participants in both arms were interviewed, more intervention participants (*n* = 15) were interviewed than control participants (*n* = 5).

An additional limitation was that the oldest participant was 61 years old and so our sample did not include those in older age groups. This likely reflects the online advertising approach adopted, although the mean age of participants in the present trial aligns closely with digital cessation trials that recruited offline.^28^ Other digital cessation app trials have also found low representation of those aged over 65 when recruiting through Facebook and Google^51^ and through app stores.^52^ Nevertheless, working on approaches to increase the age diversity of research participants would be valuable in future work. We were also unsuccessful in reaching our target for the proportion of participants who were classed as the lowest socioeconomic status category and unable to increase this using advert targeting, which will require further efforts in a future trial.

A further limitation was that this trial was undertaken during an unprecedented time of changed habits and routines due to the coronavirus disease 2019 pandemic. Participants reported reduced movement due to lockdown and similar measures to reduce movement outside of the home. It is likely this affected the exposure and time spent in different smoking locations and consequently the app’s ability to deliver context-specific support. Furthermore, the app’s use of geofencing to specify locations has potential for measurement error which can cause false negatives, where no support message is sent because of uncertainty as to whether an individual is within a geofence, due to poor location accuracy.

### Conclusions

The primarily automated trial design and processes for evaluating Quit Sense were feasible and enabled successful trial delivery within anticipated timeframes, cost, and participation rates. Evidence was consistent with Quit Sense leading to a higher rate of biochemically verified abstinence relative to usual care and all end-of-trial abstinence measures favored the app and represented clinically meaningful effects. Improving saliva sample response rates and sample diversity through advert targeting are two areas for improvement in a definitive trial, which is warranted by the findings from this feasibility trial.

## Supplementary Material

A Contributorship Form detailing each author’s specific involvement with this content, as well as any supplementary data, are available online at https://academic.oup.com/ntr.

ntad032_suppl_Supplementary_Material

ntad032_suppl_Supplementary_Figure_1

## Data Availability

The data underlying this article will be shared on reasonable request to the corresponding author.
