# Supplementary material for: An Automated, Online Feasibility Randomized Controlled Trial of a Just-In-Time Adaptive Intervention for Smoking Cessation (Quit Sense)
Source: Nicotine Tob Res. 2023 Apr 14;25(7):1319–29. doi: 10.1093/ntr/ntad032 (PMC10256891; doi:10.1093/ntr/ntad032)
Supplement: ntad032_suppl_Supplementary_Material [file ntad032_suppl_supplementary_material.docx]

**Supplementary figures and tables for article “An automated, online feasibility randomised controlled trial of a Just-In-Time Adaptive Intervention for smoking cessation (Quit Sense)”**

Supplementary figure 1 Quit Sense smartphone app intervention logic model (separate .pptx file)

Supplementary figure 2 Flow diagram of advert reach, engagement and enrolment rates and costs

Supplementary table 1 Sensitivity analysis assessing between-group differences in abstinence (primary outcome; self-reported abstinence as smoking no more than 5 cigarettes within the 6 month study period, validated by saliva test) at 6 months, excluding withdrawals, using exact inference

Supplementary table 2 Sensitivity analysis assessing between-group differences in abstinence (primary outcome; self-reported abstinence as smoking no more than 5 cigarettes within the 6 month study period, validated by saliva test) at 6 months, of complete cases, using exact inference

Supplementary table 3 Between-group differences in scores for mechanism of action measures at 6 weeks (non-parametric statistical tests)

Supplementary figure 2 Flow diagram of advert reach, engagement and enrolment rates and costs

**Enrolment and randomisation**

**(n=209; 11.3% of advert clicks)**

£5.82 per recruit, advert costs only

£19.20 per recruit, all costs

**Advert views (impressions)**

**(N=152,375)**

**Commercial ‘cost-per-click’ advertising campaign**

Campaign costs £2,796.00

**Banner**

**(Facebook Ads)**

Total spend £804.44

Duration 35 days

(phase #1 Nov/Dec 2020; #2 Jan 2021)

**Search-based**

**(Google AdWords)**

Total spend £412.49

Duration 35 days

(phase #1 Nov/Dec 2020; #2 Jan 2021)

**n=24,773**

**Advert clicks**

**(n=1,843; 1.2% of impressions)**

**n=195**

(13.8% of advert clicks)

£4.13 per recruit, advert costs only

£11.29 per recruit, all costs

**n=14**

(3.3% of advert clicks)

£29.46 per recruit, advert costs only

£129.32 per recruit, all costs

**n=425**

(1.7% of impressions)

**n=1,418**

(1.1% of impressions)

**n=129,602**

Supplementary table 1 Sensitivity analysis assessing between-group differences in abstinence (primary outcome; self-reported abstinence as smoking no more than 5 cigarettes within the 6 month study period, validated by saliva test) at 6 months, excluding withdrawals, using exact inference

| **Model^a^**  **N = 203** | **App group abstinence**  n (%)  **N = 100** | **Standard group abstinence**  n (%)  **N = 103** | **Odds ratio** | **95% Exact confidence Interval^b^** | **P-value** |
| --- | --- | --- | --- | --- | --- |
| Adjusted for treatment group only | 12 (12.0%) | 3 (2.9%) | 4.52 | (1.17, 25.73) | 0.025 |
| Adjusted model, including treatment group and stratification variables | 12 (12.0%) | 3 (2.9%) | 4.53 | (1.15, 26.09) | 0.026 |
| Adjusted model, including treatment group, stratification and prognostic variables | 12 (12.0%) | 3 (2.9%) | 4.45 | (1.13, 25.76) | 0.030 |

^a^Logistic regression model used, modelling the odds of abstinence, adjusted for differences of smoking rate at baseline, socioeconomic status at baseline (stratification variables), heaviness index at baseline (prognostic variable) and treatment group. This is a conservative model, including those lost to follow up (non-responders), as well as complete cases, with any ‘missing’ values set to not abstinent. Withdrawals (6 participants) are excluded as a sensitivity analysis.

^b^95% Exact confidence Interval for odds ratio.

Supplementary table 2 Sensitivity analysis assessing between-group differences in abstinence (primary outcome; self-reported abstinence as smoking no more than 5 cigarettes within the 6 month study period, validated by saliva test) at 6 months, of complete cases, using exact inference

| **Model^a^**  **N = 131** | **App group abstinence**  n (%)  **N = 65** | **Standard group abstinence**  n (%)  **N = 66** | **Odds ratio** | **95% Exact confidence Interval^b^** | **P-value** |
| --- | --- | --- | --- | --- | --- |
| Adjusted for treatment group only | 12 (18.5%) | 3 (4.5%) | 4.70 | (1.18, 27.32) | 0.023 |
| Adjusted model, including treatment group and stratification variables | 12 (18.5%) | 3 (4.5%) | 4.71 | (1.17, 27.64) | 0.025 |
| Adjusted model, including treatment group, stratification and prognostic variables | 12 (18.5%) | 3 (4.5%) | 4.88 | (1.19, 29.03) | 0.024 |
| Missing | 39 | 39 |  |  |  |

^a^Logistic regression model used, modelling the odds of abstinence, adjusted for differences of smoking rate at baseline, socioeconomic status at baseline (stratification variables), heaviness index at baseline (prognostic variable) and treatment group. Those non-responders, withdrawals and missing, remain missing.

^b^95% Exact confidence Interval for odds ratio.

Supplementary table 3 Between-group differences in scores for mechanism of action measures at 6 weeks (non-parametric statistical tests)

| **Categorical/continuous variable at 6 weeks** | **App**  **(n=71)** | **Standard**  **(n=78)** | **Statistical test/mean difference (95% CI)** | **p-value** |
| --- | --- | --- | --- | --- |
| Did not smoke tobacco in the first 2 weeks of quit attempt (or first 2 weeks of study) (lapse incidence) | 21 (29.6%) | 15 (19.2%) | *X*^2^, (1, 149) = 2.17 | 0.141 |
| Did not smoke any tobacco in the first month of quit attempt (or first month of study) (lapse incidence) | 11 (15.5%) | 7 (9.0%) | *X*^2^, (1, 149) = 1.49 | 0.223 |
| Strength of the urge to smoke (SUTS)^a^   - No urges - Slight urges - Moderate urges - Strong urges - Very strong urges - Extremely strong urges | 12 (16.9%)  9 (12.7%)  29 (40.9%)  7 (9.9%)  8 (11.3%)  6 (8.5%) | 3 (3.9%)  20 (25.6%)  22 (28.2%)  16 (20.5%)  9 (11.5%)  8 (10.3%) | Mann-Whitney U = 5016.00 | 0.228 |
| Frequency of the urge to smoke (FUTS) ^a^   - Not at all - A little of the time - Some of the time - A lot of the time - Almost all the time - All the time | 10 (14.1%)  15 (21.1%)  17 (23.9%)  12 (16.9%)  9 (12.7%)  8 (11.3%) | 6 (7.7%)  21 (26.9%)  17 (21.8%)  18 (23.1%)  9 (11.5%)  7 (9.0%) | Mann-Whitney U = 5267.50 | 0.825 |
| Self-efficacy average score (five-point scale) | 1.89 (1.26) | 1.71 (1.11) | Mann-Whitney U = 5550.50 | 0.391 |
| WISDM Automaticity subscale score (seven-point scale) | 3.56 (2.26) | 3.83 (2.30) | Mann-Whitney U = 5153.50 | 0.512 |
| Lapse prevention average score^b^ | 1.38 (0.57) | 1.46 (0.62) | -0.07 (-0.26, 0.12) | 0.455 |
| Lapse prevention subscale (avoidance) average score^b^ | 1.23 (0.73) | 1.33 (0.68) | -0.10 (-0.33, 0.13) | 0.394 |
| Lapse prevention subscale (coping) average score^b^ | 1.47 (0.56) | 1.53 (0.67) | -0.06 (-0.26, 0.14) | 0.560 |
| WISDM Cue exposure/Associative processes subscale score | 4.38 (1.55) | 4.24 (1.52) | 0.14 (-0.36, 0.64) | 0.577 |

^a^ Scale variable with mean not calculated; ^b^Mean score on four response options (1=not used at all, 2=used 1-5 times, 3=used 6-10 times, 4=used more than 10 times) for 20 strategies;
