# Supplementary material for: An Automated, Online Feasibility Randomized Controlled Trial of a Just-In-Time Adaptive Intervention for Smoking Cessation (Quit Sense)
Source: Nicotine Tob Res. 2023 Apr 14;25(7):1319–29. doi: 10.1093/ntr/ntad032 (PMC10256891; doi:10.1093/ntr/ntad032)
Supplement: ntad032_suppl_Supplementary_Figure_1 [file ntad032_suppl_supplementary_figure_1.pptx]

## Slide 1
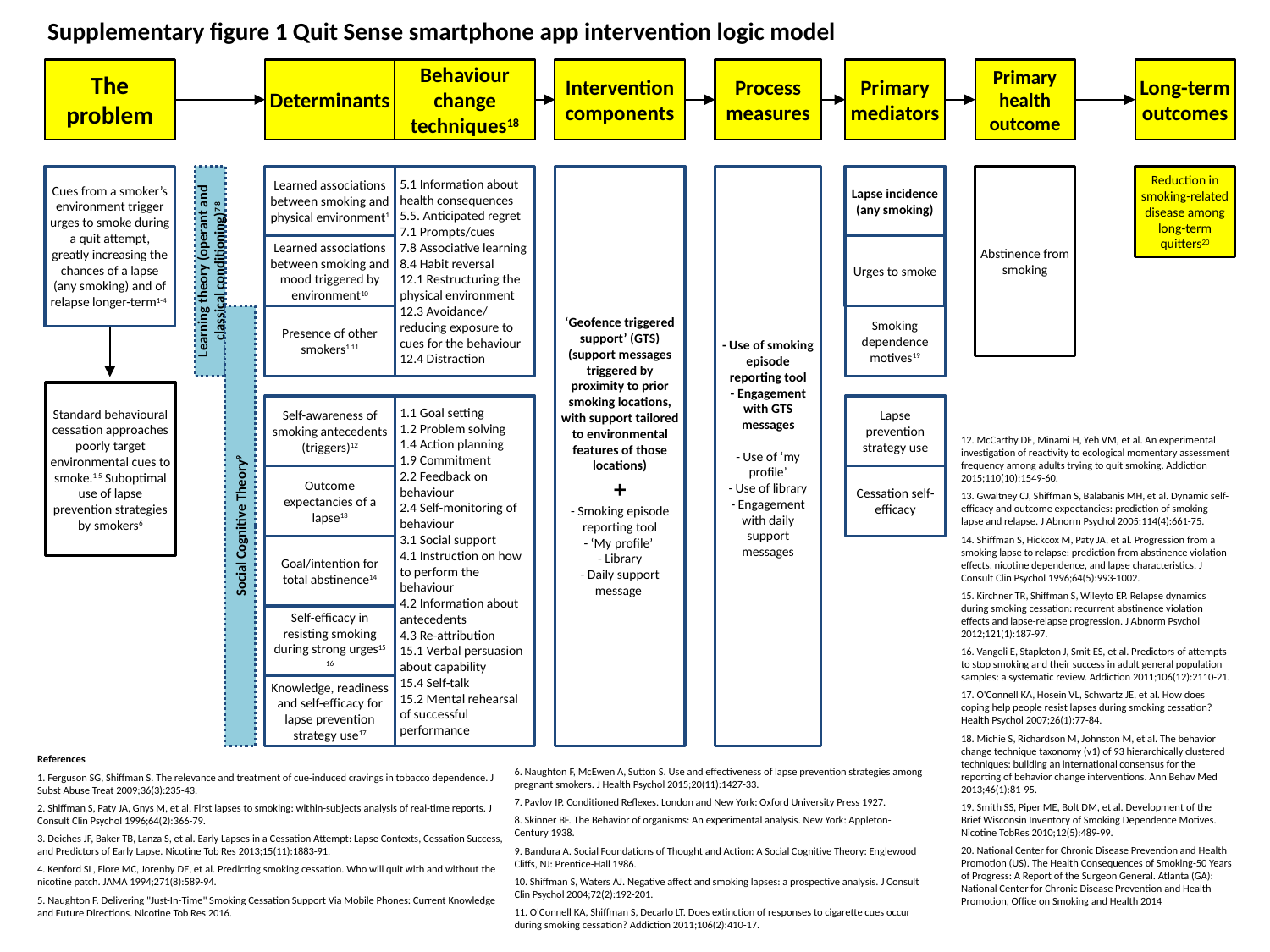

Supplementary figure 1 Quit Sense smartphone app intervention logic model
The problem
Determinants
Behaviour change techniques18
Intervention components
Process measures
Primary mediators
Primary health outcome
Long-term outcomes
Cues from a smoker’s environment trigger urges to smoke during a quit attempt, greatly increasing the chances of a lapse (any smoking) and of relapse longer-term1-4
Learned associations between smoking and physical environment1
5.1 Information about health consequences
5.5. Anticipated regret
7.1 Prompts/cues
7.8 Associative learning
8.4 Habit reversal
12.1 Restructuring the physical environment
12.3 Avoidance/ reducing exposure to cues for the behaviour
12.4 Distraction
‘Geofence triggered support’ (GTS)(support messages triggered by proximity to prior smoking locations, with support tailored to environmental features of those locations)
+
- Smoking episode reporting tool
- ‘My profile’
- Library
- Daily support message
- Use of smoking episode reporting tool
- Engagement with GTS messages
- Use of ‘my profile’
- Use of library
- Engagement with daily support messages
Lapse incidence (any smoking)
Abstinence from smoking
Reduction in smoking-related disease among long-term quitters20
Urges to smoke
Learned associations between smoking and mood triggered by environment10
Learning theory (operant and classical conditioning)7 8
Presence of other smokers1 11
Smoking dependence motives19
Standard behavioural cessation approaches poorly target environmental cues to smoke.1 5 Suboptimal use of lapse prevention strategies by smokers6
Self-awareness of smoking antecedents (triggers)12
1.1 Goal setting
1.2 Problem solving
1.4 Action planning
1.9 Commitment
2.2 Feedback on behaviour
2.4 Self-monitoring of behaviour
3.1 Social support
4.1 Instruction on how to perform the behaviour
4.2 Information about antecedents
4.3 Re-attribution
15.1 Verbal persuasion about capability
15.4 Self-talk
15.2 Mental rehearsal of successful performance
Lapse prevention strategy use
12. McCarthy DE, Minami H, Yeh VM, et al. An experimental investigation of reactivity to ecological momentary assessment frequency among adults trying to quit smoking. Addiction 2015;110(10):1549-60.
13. Gwaltney CJ, Shiffman S, Balabanis MH, et al. Dynamic self-efficacy and outcome expectancies: prediction of smoking lapse and relapse. J Abnorm Psychol 2005;114(4):661-75.
14. Shiffman S, Hickcox M, Paty JA, et al. Progression from a smoking lapse to relapse: prediction from abstinence violation effects, nicotine dependence, and lapse characteristics. J Consult Clin Psychol 1996;64(5):993-1002.
15. Kirchner TR, Shiffman S, Wileyto EP. Relapse dynamics during smoking cessation: recurrent abstinence violation effects and lapse-relapse progression. J Abnorm Psychol 2012;121(1):187-97.
16. Vangeli E, Stapleton J, Smit ES, et al. Predictors of attempts to stop smoking and their success in adult general population samples: a systematic review. Addiction 2011;106(12):2110-21.
17. O'Connell KA, Hosein VL, Schwartz JE, et al. How does coping help people resist lapses during smoking cessation? Health Psychol 2007;26(1):77-84.
18. Michie S, Richardson M, Johnston M, et al. The behavior change technique taxonomy (v1) of 93 hierarchically clustered techniques: building an international consensus for the reporting of behavior change interventions. Ann Behav Med 2013;46(1):81-95.
19. Smith SS, Piper ME, Bolt DM, et al. Development of the Brief Wisconsin Inventory of Smoking Dependence Motives. Nicotine TobRes 2010;12(5):489-99.
20. National Center for Chronic Disease Prevention and Health Promotion (US). The Health Consequences of Smoking-50 Years of Progress: A Report of the Surgeon General. Atlanta (GA): National Center for Chronic Disease Prevention and Health Promotion, Office on Smoking and Health 2014
Cessation self-efficacy
Outcome expectancies of a lapse13
Social Cognitive Theory9
Goal/intention for total abstinence14
Self-efficacy in resisting smoking during strong urges15 16
Knowledge, readiness and self-efficacy for lapse prevention strategy use17
References
1. Ferguson SG, Shiffman S. The relevance and treatment of cue-induced cravings in tobacco dependence. J Subst Abuse Treat 2009;36(3):235-43.
2. Shiffman S, Paty JA, Gnys M, et al. First lapses to smoking: within-subjects analysis of real-time reports. J Consult Clin Psychol 1996;64(2):366-79.
3. Deiches JF, Baker TB, Lanza S, et al. Early Lapses in a Cessation Attempt: Lapse Contexts, Cessation Success, and Predictors of Early Lapse. Nicotine Tob Res 2013;15(11):1883-91.
4. Kenford SL, Fiore MC, Jorenby DE, et al. Predicting smoking cessation. Who will quit with and without the nicotine patch. JAMA 1994;271(8):589-94.
5. Naughton F. Delivering "Just-In-Time" Smoking Cessation Support Via Mobile Phones: Current Knowledge and Future Directions. Nicotine Tob Res 2016.
6. Naughton F, McEwen A, Sutton S. Use and effectiveness of lapse prevention strategies among pregnant smokers. J Health Psychol 2015;20(11):1427-33.
7. Pavlov IP. Conditioned Reflexes. London and New York: Oxford University Press 1927.
8. Skinner BF. The Behavior of organisms: An experimental analysis. New York: Appleton-Century 1938.
9. Bandura A. Social Foundations of Thought and Action: A Social Cognitive Theory: Englewood Cliffs, NJ: Prentice-Hall 1986.
10. Shiffman S, Waters AJ. Negative affect and smoking lapses: a prospective analysis. J Consult Clin Psychol 2004;72(2):192-201.
11. O'Connell KA, Shiffman S, Decarlo LT. Does extinction of responses to cigarette cues occur during smoking cessation? Addiction 2011;106(2):410-17.
